# Resilient reefs may exist, but can larval dispersal models find them?

**DOI:** 10.1371/journal.pbio.2005964

**Published:** 2018-08-22

**Authors:** Michael Bode, Lance Bode, Severine Choukroun, Maurice K. James, Luciano B. Mason

**Affiliations:** 1 School of Mathematical Sciences, Queensland University of Technology, Brisbane, Australia; 2 Australian Research Council (ARC), Centre of Excellence in Coral Reef Studies, James Cook University, Townsville, Australia; 3 College of Science and Engineering, James Cook University, Townsville, Australia

The Great Barrier Reef (GBR) is an interconnected system of thousands of coral reefs and shoals spanning more than 2,000 km of the eastern Australian coastline. Anthropogenic pressures—principally climate change—pose an existential threat to this iconic marine ecosystem [1]. Management actions are urgently required to halt and reverse the degradation, but the GBR’s enormous size creates logistical and budgetary challenges.

In a recent research article, Hock and colleagues [2] offer a solution to this predicament: They argue that a tiny fraction of the GBR’s reefs—fewer than 1%—act as its ‘life-support system’ [3]. The reefs are primarily identified by their larval connectivity, the movement of juvenile individuals between reefs on ocean currents. A dispersive larval stage is common to many species on coral reefs—notably fishes and reef-building corals—and is an essential source of new recruits following mortality and disturbance. Unfortunately, because larvae are difficult and expensive to follow during their pelagic dispersal phase, empirical data are not available at management-relevant scales [4]. Instead, Hock and colleagues base their decisions on ‘biophysical models’—computer simulations that integrate individual-based models of larval behaviour with hydrodynamic ocean current models. In recent years, biophysical models have become a standard tool for investigating the effects of larval connectivity on marine ecology, evolution, and conservation [4–6].

Because their biophysical model predicts that a small number of reefs contribute disproportionately to the GBR’s persistence and resilience, Hock and colleagues claim that these reefs should be a primary focus of management resources. In this comment, we show that the identity of these reefs should be treated with caution, since different numerical models of larval connectivity select different reefs as priorities. More generally, we argue that the current generation of biophysical models should not be used to guide management actions at the scale of individual reefs.

The last decade has seen rapid advances in the sophistication of biophysical models. High-performance computing allows these models to simulate billions of larval releases across thousands of kilometres, while their biological components now incorporate experimentally measured sensory and swimming capabilities. However, despite these strengths, accurate numerical modelling of shallow coastal flow fields with highly variable bathymetry—conditions typical of coral reefs—remains an immense challenge. Critical near-reef hydrodynamics often vary at substantially smaller scales (1 m–1,000 m) than the resolution of biophysical models (100 m–10,000 m; [7]). Such difficulties are exacerbated by uncertainty about the parameters that describe larval development, behaviour, and mortality, as well as adult spawning behaviour [6]. These limitations have been debated extensively [8]; given this level of uncertainty, we have strong reservations about whether existing biophysical models are accurate enough to identify reefs that have particular larval connectivity characteristics or to identify the type of life-support system highlighted in [2].

To test this question, we repeated Hock and colleagues’ analyses using a different model of larval dispersal on the GBR. While not identical, the two models are functionally equivalent: comparable in complexity, resolution, and scale. They are both based on hydrodynamic models of the entire GBR and predict the dispersal of numerous species from multiple larval spawning events across many years (see S1 Text). We used the predictions of this alternative model to reclassify ‘key source reefs’, a critical step in Hock and colleagues’ methodology. They identified 545 key sources, which they define as reefs that exhibit (i) high out degree, (ii) high node strength, (iii) a large number of strong connections, (iv) strong connections to other key sources, and (v) high connectedness. Precise mathematical definitions are detailed in [2].

Despite the similarities between the two biophysical models, they identify very different key source reefs (Fig 1). Both models prioritise reefs in the mid and outer shelf, but the alternative model chooses reefs throughout the central GBR and in the inner-shelf Whitsunday region, where Hock and colleagues found no key sources. Overall, only 32% of the key sources prioritised by our alternative model were also highlighted by Hock and colleagues. Moreover, a substantial proportion (19%) of the key sources identified in [2] are among the worst reefs according to the alternative model, consistently delivering the lowest performance according to all five criteria.

**Fig 1:**
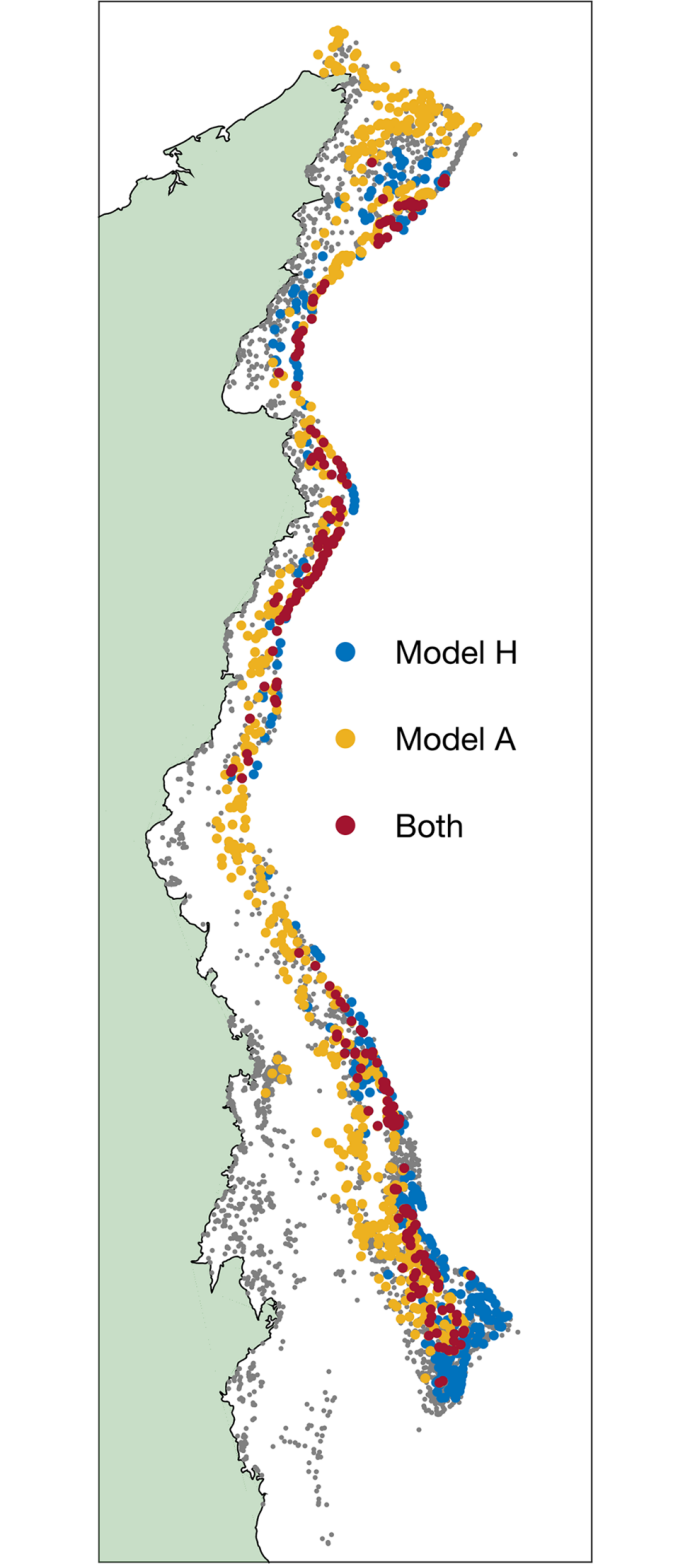
‘Key source reefs’ on the GBR, identified by two alternative larval dispersal models. Reefs identified as key sources by Hock and colleagues. [2] (Model H) only are shown in blue. Reefs identified by the alternative model (Model A) only are shown in yellow. Key sources in both models are identified by red circles. The Australian coast is shown in green. GBR, Great Barrier Reef.

It is important to note that we are not arguing that our alternative biophysical model—or the reefs that it selects—is superior to Hock and colleagues’ model. Both are based on advanced hydrodynamic models and describe larval dispersal in the same coral reef ecosystem. Different assumptions were made in their construction (see S1 Text), but both sets of choices are common and defensible in biophysical modelling. The fact that two advanced models identify different priorities suggests that the current generation of biophysical models do not make consistent predictions about larval dispersal patterns, at least not at the scale of individual reefs.

We draw four conclusions from the differences in model predictions. First, Hock and colleagues state that their key sources ‘offer a tangible and feasible set of intervention points’ for managers [2,3]. Our results suggest that GBR managers should view this set of reefs with caution, since they are heavily dependent on a specific biophysical model. Second, we believe that this same caution should extend across the marine biome. Researchers are increasingly using biophysical larval dispersal models to identify priorities at very fine spatial scales [9,10]; we ourselves have not been immune to this temptation [5,11]. Since managers are likely to continue to seek guidance at the scale of individual reefs—this is a common size for marine reserves, for example—it is important that any such recommendations come with strong caveats. Third, future research should aim to better understand the relationship between biophysical model uncertainty and spatial scale. Ideally, we would directly compare biophysical model predictions to observations of larval dispersal, but this is beyond the reach of current empirical data. In the interim, comparisons among biophysical models, such as the one offered here, will provide some insight into their ability to support robust decisions at different scales. Although our results show that models disagree on the strength of connectivity between individual reefs, their predictions may correspond at some larger scale (e.g., groups of reefs [12]). Finally, regardless of the agreement between different biophysical models, decisions that concern larval dispersal should be based on the predictions of multimodel ensembles. Extensive evidence from other nonlinear systems (e.g., meteorology and climatology) shows that ensemble forecasts are more accurate and more robust than any single model [13].

## Supporting information

S1 Text Full description of the alternative biophysical model used in the comparison. Includes a detailed comparison of the two models.
(DOCX)
