# Supplementary material for: Resilient reefs may exist, but can larval dispersal models find them?
Source: PLoS Biol. 2018 Aug 22;16(8):e2005964. doi: 10.1371/journal.pbio.2005964 (PMC6104909; doi:10.1371/journal.pbio.2005964)
Supplement: S1 Text — Includes a detailed comparison of the two models. (DOCX) [file pbio.2005964.s001.docx]

**Supporting information *S1 Text***

**Title: Resilient reefs may exist, but can larval dispersal models find them?**

Authors: Bode, M., Bode, L., Choukroun, S., James, M.K., Mason, L.B.

We classified “key source reefs” as described in the original article, but using larval connectivity predictions from an alternative biophysical model of the Great Barrier Reef (GBR). For the specific mathematical definitions of the criteria used to identify key source reefs, see *Materials and Methods* in Hock et al. (2017).

*Description of the alternative biophysical model*

Water currents were constructed using a numerical model of hydrodynamic flow in the coastal shelf and adjoining regions of the Coral Sea. A continuous current record was simulated between years 1996 and 2002 inclusive. Flows were calculated using a two-dimensional, depth-integrated current field for the reef-shelf complex, at a spatiotemporal resolution of 1 nautical mile, and 0.5 hours. A uniform spatial resolution ensures that near-reef dynamics (critical for larval retention, and thus the definition of key sources) are modelled consistently across the system. To account for flow changes in the immediate vicinity of reefs, we used a parameterisation scheme that accounts for the sub-grid-scale hydrodynamics resulting from the specific geomorphology of coral reef systems (Bode & Mason 1994). The currents were forced by a combination of tides, wind (derived from Australian Bureau of Meteorology LAPS analysis), and oceanic influences from the East Australia Current (including data on seasonal variability in its strength and location). Tides were calibrated using coastal and reef based tidal analyses, and low-frequency currents on the shelf were validated by three years of data from TEACS moorings near Lark Reef.

Lagrangian larval dispersal events were simulated using an individual-based model which accounted for larval behaviour and development. Releases were modelled from each of 2,175 reefs between 9^o^S and 24.5^o^S, with reef locations and geometry taken from the Great Barrier Reef Marine Park Authority. Larvae were released from the outer perimeter of each reef, at a density of 3,900 km^-1^. Spawning was assumed to occur daily at each sunset during the summer spawning season, from September 16 until February 18, and the results were amalgamated into annual connectivity matrices. Once released, the larvae began a precompetent period of either 7 days or 14 days, during which time they were dispersed as passive, neutrally-buoyant particles in the mid-water column, moving with the depth-integrated current. After this period they became active, and were able to settle on the first reef that came within a settlement radius of either 1 km or 4 km. During both their precompetent and competent dispersive phases, larval trajectories were calculated using a second-order Runge Kutta algorithm, where instantaneous velocity is a linear interpolation of the velocities in adjacent grid points. Random diffusion was not added to the model to simulate sub-grid-scale horizontal eddy diffusion, as the model resolution was sufficiently high to allow substantial endogenous eddy formation and horizontal mixing, particularly in the vicinity and lee of reefs. During dispersal, larvae experienced mortality regimes of either 18% per day, or 18% per day and 90% per day when they spent precompetent time over a reef. Larvae were removed from the model, assumed dead, if they had not settled after either 3 weeks, or 6 weeks of competency.

In both Hock et al. (2017) and this alternative model, the larval parameters (e.g., competency length) were not chosen to reproduce any particular species. Instead, they were varied in exhaustive combination to create multiple “pseudospecies” with different dispersal characteristics, in an attempt to capture a range of possible species. Additional published details about the numerical model, its biological components, and analyses of its predictions can be found in Bode & Mason (1994), James *et al*. (2002), and Bode *et al*. (1997; 2012). In total, combinations of these factors created 112 different connectivity matrices (7 years x 2 precompetent periods x 2 competent periods x 2 settlement zones x 2 mortality regimes) for use in the classification of “key sources”.

*Comparison between the two models*

Both biophysical models predict larval dispersal patterns over multiple years, across the whole GBR. We hereafter refer to the Hock et al. (2017) model as **H**, and the alternative model described above as **A**. The hydrodynamic components of both models are based around numerical finite difference schemes, with comparable spatiotemporal resolutions (**H** = 4 km & 1 hour; **A** = 1.6 km & 0.5 hour). Both release large numbers of larvae from each source reef at each dispersal event (**H** = $1\times{10}^{4}$ larvae; **A** = $4\times{10}^{5}$ larvae), which are passive during a precompetent phase, after which they become competent and can settle on reefs that come within a sensory radius. Both models simulate mortality at various rates during the dispersal phase.

The two models apply different definitions of “reef” (**H** defines 3,806 reefs, **A** defines 2,175 reefs). This difference is partly due to different choices about what constitutes a single “reef”. The Great Barrier Reef Marine Park Authority currently recognises 4,480 separate elements of reef habitat on the GBR, and the two models use different rules to group these noncontiguous habitat elements into individual patch reefs. Model **A** also excludes elements of reef habitat with minimum depths greater than 10 m, on the premise that such deeper-water reef habitat has different species assemblages and environmental characteristics to shallow-water reef habitat. When comparing between the two models, we matched each patch reef in model **H** to the closest patch reef in model **A**, but only if their centroids were within 4 km of each other (the spatial resolution of model **H**). This meant that some of the reefs in **A** were matched to multiple reefs in model **H**. Based on this definition, we found that 908 patch reefs in **H** (23%) could not be matched to a reef in **A**, and that 301 patch reefs in **A** (14%) were not found in model **H**. We excluded unmatched reefs in both sets from the analyses, but also found that the decision to include or exclude these reefs did not substantially change the proportional agreement between the two models.

There are differences – hydrodynamic, biological and temporal – between the two biophysical models. These include different years (**A**: 1996 – 2002; **H**: 2008 – 2013), and different pseudospecies (**A** = 16 spp; **H** = 13 spp). In comparing the two models, we believe that its reasonable to expect two forms of agreement between their sets of key source reefs. First, the reefs chosen as key sources by model **H** should frequently be key source reefs according to model **A**. Second, a given reef should not be considered a high priority by one model, and a low priority by the other model. We expect this agreement despite the fact that the two models disagree in three separate ways: different years, different species, and different modelling decisions. There are two reasons we expect agreement. First, Hock et al. specifically argue that the identity of key source reefs is robust to the first two differences – years and species. As they describe them, key sources are intended to support the recovery of a wide range of coral reef organisms, under a wide range of conditions. This is why the authors used 208 different connectivity matrices to identify them, representing multiple species and multiple years. While numerous, their matrices only cover a subset of climatic and hydrodynamic conditions, and of the dispersal characteristics of coral reef organisms. If the key sources are indeed robust, they should offer the same benefits to the species and years simulated by model **A**. Second, the key sources should also be robust to differences in the assumptions and techniques used to model ocean currents and larval behaviour. For example, model **H** is based on a three-dimensional baroclinic model, while model **A** is based on a two-dimensional depth-integrated model. Different choices were made in the construction of biophysical models **H** and **A**, but both sets of choices represent common and defensible decisions in biophysical modelling. If management priorities vary substantially with such choices, then the resulting recommendations cannot be considered robust.

**References cited**

Bode, L. & Mason, L. B. (1994). Application of an implicit hydrodynamic model over a range of spatial scales. In Computational techniques and applications: CTAC93 (ed. D. Stewart), pp. 112–121. Singapore: World Scientific.

Bode, L., Mason, L. B., & Middleton, J. H. (1997). Reef parameterisation schemes with applications to tidal modelling. Progress in Oceanography, 40(1-4), 285-324.

Bode, M., Armsworth, P. R., Fox, H. E., & Bode, L. (2012). Surrogates for reef fish connectivity when designing marine protected area networks. Marine Ecology Progress Series, 466, 155-166.

Hock, K., Wolff, N. H., Ortiz, J. C., Condie, S. A., Anthony, K. R., Blackwell, P. G., & Mumby, P. J. (2017). Connectivity and systemic resilience of the Great Barrier Reef. PLoS biology, 15(11), e2003355.

James, M. K., Armsworth, P. R., Mason, L. B., & Bode, L. (2002). The structure of reef fish metapopulations: modelling larval dispersal and retention patterns. Proceedings of the Royal Society of London B: Biological Sciences, 269(1505), 2079-2086.
